# Obscured Rhythms: Using Rhythmanalysis to Understand How Symptoms Associated With Menopause Shape Everyday Experiences of Physical Activity

**DOI:** 10.1111/1467-9566.70269

**Published:** 2026-09-30

**Authors:** Cassandra Phoenix, Meridith Griffin, Erica V. Bennett

**Affiliations:** ^1^ Department of Sport & Exercise Sciences Institute of Medical Humanities Durham University Durham UK; ^2^ Department of Health, Ageing & Society McMaster University Hamilton Ontario Canada; ^3^ School of Kinesiology The University of British Columbia Vancouver British Columbia Canada

## Abstract

Physical activity (PA) is frequently promoted as a means of managing symptoms associated with the menopause transition (MT), yet little is known of how the embodied, emotional and temporal disruptions of menopause influence the experiences of, and possibilities for, movement. Taking inspiration from French sociologist Henri Lefebvre's theory of ‘rhythmanalysis’ and Jennifer Mason's facet methodology approach, we draw on qualitative survey responses from 249 women to examine how symptoms associated with the MT interrelate with space and time to shape everyday experiences of PA. Our findings highlight how symptoms intertwine with seasonal, hormonal and circadian cycles to reconfigure everyday tempos of energy, rest and aspirations to move more. These shifting rhythms often constrain participation in PA, but also prompt creative adjustments including new pacing strategies, altered routines and re‐attunements to bodily fluctuation. Our rhythmanalytic approach challenges binary framings of PA as either hindered or facilitated, offering instead a textured account of how movement, stillness and slowing are negotiated during the MT. These original insights contribute to embodied and critical perspectives on health, demonstrating the value of more flexible, rhythmic and context‐sensitive approaches to promoting movement through the MT.

## 1 Introduction

Described by the British Menopause Society as ‘a major life event affecting all women, in a variety of ways, both short and long term’, menopause affects around 50% of the global population (Currie et al. 2024, 1). Once whispered about in private, it has recently surged into public consciousness, carried by podcasts, celebrity memoirs and a proliferating wellness industry promising to ‘balance hormones’ and ‘reclaim vitality’. In the UK and beyond, this cultural moment–dubbed a ‘menopausal turn’ (Jermyn 2023; Orgad and Rottenberg 2024)–has prompted new conversations about bodies, gender and midlife, while simultaneously producing new regimes of expertise, visibility and prescription. Yet to date, the lived, embodied experiences of moving through menopause–the fatigue, the sleepless nights, the aching joints the recalibrations of energy and confidence—often remain obscured beneath the noise of advice and advocacy.

Biologically, the menopause is marked by the end of monthly menstruation commonly experienced between the ages of 45 and 55 years. Although typically a natural part of ageing, menopause can also be a consequence of surgical or medical procedures. Perimenopause can last for several years and refers to the phase of fluctuating hormone levels and irregular menstrual cycles preceding menopause, ending 1 year after the final menstrual period (marking the start of ‘post‐menopause’) (World Health Organization 2024). In this paper, we use the term *menopause transition* (MT) as an overall term to cover peri to post‐menopause.

Hickey et al. (2022) assert that to describe this life event in biomedical terms alone is to miss the layered and uneven ways it unfolds across time, space and circumstance. Indeed, for some, the MT is a subtle shift; for others, a seismic interruption. These differences reflect not only biology but also the social, cultural and political rhythms of midlife: the pressures of work, caregiving and gendered expectations of productivity (Wood, McCarthy, Pitt, Randle, Arnot, et al. 2025a). Commonly reported symptoms such as hot flashes, insomnia, joint pain and brain fog are well documented but many women are still not receiving adequate information and support (Barber and Charles 2023). Management options include menopause hormone therapy (MHT) (previously referred to as Hormone Replacement Therapy (HRT); a term still often utilised) and/or lifestyle medicine in which physical activity (PA) represents a key component. Indeed, across medical, fitness and media landscapes, the role of regular PA features highly in facilitating positive psychological and social health outcomes, maintaining bone and heart health and protecting against the decline and risk associated with hormonal change during the MT (Hybholt 2022; Hulteen et al. 2023). Menopause workouts, targeted online programs, and motivational slogans abound.

While this narrative around PA and MT is compelling, several important components to the story are often glossed over. Decades of underinvestment in women's health have left significant gaps in our understanding of how changing hormonal profiles shape lived experiences of physical activity (McNulty, Lane, et al. 2024; McNulty, Olenick, et al. 2024). Furthermore, evidence for the efficacy of PA in alleviating specific menopausal symptoms remains inconsistent (A. Thomas and Daley 2020). Meanwhile, the global status of PA is such that structural, cultural and emotional constraints—lack of time, stigma, safety concerns, low confidence and caring responsibilities—continue to limit women's access to and enjoyment of PA in general (World Health and Organization 2022). This can be exacerbated during the MT (McNulty, Lane, et al. 2024; McNulty, Olenick, et al. 2024; Wood, McCarthy, Pitt, Randle, Arnot, et al. 2025a). Hybholt and Spotswood (2025) have shown how just the emotional labour required to prioritise active leisure during midlife can itself be exhausting; a scenario further exemplified by Wood, McCarthy, Pitt, Randle and Thomas (2025) as they discuss the concept of ‘midlife collision’ to show how the MT often coincides with profound personal challenges and gendered expectations of midlife women. These tensions can become amplified by the growing chorus of ‘expert voices’ that frame menopause and movement through the language of optimisation–urging women to ‘harness their hormones’ and ‘take control’ (Nappi et al. 2024). Such narratives risk flattening experience and reinforcing narrow ideals of productivity and self‐discipline (Badr 2022). In doing so, they can silence the textured, contingent and sometimes contradictory ways women inhabit and make sense of their changing bodies (Lyons and Griffin 2003).

This paper provides an ‘overdue and welcome’ contribution to knowledge by foregrounding everyday experiences of the MT (S. L. Thomas et al. 2024, 3). Rather than seeking to ascertain what effect PA has on women's menopause symptoms, instead we ask, *how do women feel the symptoms they associate with their MT impact their experiences of physical activity*? Switching the focus in this way invites a departure from default ‘exercise is medicine’ framings, which can inadvertently assume that exercise is good/positive for everyone (see Cairney et al. 2018). In so doing, space is created for more complex entanglements of embodiment, emotion and environment to potentially surface and be explained, thereby advancing current understandings of movement through menopause.

## 2 Research Design and Methods

Like much qualitative work in health research, the focus of our research was on the meaning of a phenomenon and accordingly, was situated within an interpretivist paradigm (Green and Thorogood 2018). From this perspective, all knowledge claims are thus partial, contingent, historically, culturally and contextually bound (Sparkes 1992). Our interest was in women's interpretation of their symptoms and the meanings these held for how they planned, approached, engaged in or even avoided physical activity.^1^ Data was gathered using an online qualitative survey, where participants were invited to provide typed responses to a series of open‐ended questions about their menopause symptoms and physical activity (see Table 1). Prompts were included in the survey instructions and following the questions, encouraging respondents to provide as much detail as possible using their own words and to include illustrative examples where it felt helpful.

**TABLE 1:** Open‐ended survey questions.

1.	Please tell us about how your menopausal symptoms impact on your experiences of physical activity.
2.	What (if anything) do you find yourself needing to consider in relation to your menopause symptoms when engaging, or planning to engage in physical activity?
3.	What do you wish people knew about the impact of menopausal symptoms on physical activity, but is often not talked about?
4.	What would you say is the one symptom that impacts your day‐to‐day life over and above others you may experience, and why?
5.	Is there anything else that you think is important for us to know about this topic, but haven't had the opportunity to tell us so far?
	Questions (optional) to collect demographic details.

S. L. Thomas et al. (2024, 1) maintain that ‘[R]igorous online qualitative surveys that are grounded in qualitative interpretivist values offer a range of unique benefits for public health researchers, knowledge users and research participants’. Moreover, this method is well‐suited to researching sensitive subjects where ‘felt anonymity’ is high, potentially facilitating less inhibited responses from participants (Terry and Braun 2017). Participants may feel an increased sense of autonomy, as they decide on the length, timing and location of their contributions. These values were pertinent to the current study, given the highly personal and for some, embarrassing, nature of the topic given its historical framing around secrecy and taboo.

As we designed the survey, in the UK, we consulted with a pre‐existing research partner group (aligned with the Institute of Medical Humanities, Durham University) who deliberated about the survey questions via virtual discussions. Their feedback led us to refine our demographic categories and add a question asking if participants were taking medication and/or receiving treatment for the menopause. In Canada, the survey was reviewed by three critical peers whose feedback resulted in the refinement of demographic categories pertaining to gender, sexual orientation and ethnicity (see Table S1).

Criteria for participation included being aged 18 years+, and experiencing symptoms understood as being associated with the MT. This lower inclusion age meant that women experiencing early menopause would not be excluded from the study. Aware that severe symptoms can often deter engagement in PA (and our interest in this phenomenon extended to include why and how), current level of PA was collected but not used as an inclusion criteria. Prior to data generation, ethical approval was gained from Durham University Department of Sport and Exercise Sciences Research Ethics Committee, the University of British Columbia's Behavioural Research Ethics Board, and the McMaster Research Ethics Board.

A dissemination/recruitment strategy aimed at reaching a diverse audience was developed in partnership with our advisory board and critical peers. Different distribution pathways were pursued including: via dedicated project accounts on social media (e.g., Instagram); Facebook posts (personal and research institution accounts) tagging online menopause groups; emails/phone communication and social media posts with and to community groups (/group leaders) focusing on menopause in queer, trans/nonbinary, Indigenous and Black communities, as well as community serving organisations; email listservs (academic and non‐academic networks).

In total, 249 people responded to the survey. None of the survey questions (except those relating to informed consent) were marked as compulsory, allowing participants to contribute as they wished. All provided a written response to each of the five questions, resulting in 1245 separate text entries. While inevitably, a small number of these involved short sentences or single word answers, we were struck by the rich descriptions of personal narratives, emotional reflections and nuanced descriptions of lived experience provided by most participants. That said, like all methods, qualitative surveys have some limitations. For example, being unable to ask follow‐up questions (as one might during an in‐person interview), meant that some responses would always lack context, ultimately shaping both the depth and scope of some of the insights produced.

The samples were predominantly white/European, cis/heterosexual, able‐bodied and University educated (75%). The majority were aged 45–59 years, over half were receiving treatment for menopause symptoms (e.g., MHT, complementary therapies), and 51% self‐reported participating in 150+ min of physical activity per week (see Table 2; see Table S1 for full breakdown of participant details). Of note, 2% (*n* = 6) of participants indicated they were aged 30–39 years (4 self‐defined as perimenopausal, 1 as early menopause and 1 undergoing medical tests for early menopause). Their responses did not differ markedly from the older participants and were included into the main analysis.

**TABLE 2:** Selected demographic details of research participants.

Participant characteristic	No. of participants	% Participants
Responding to survey circulated by UK collaboration hub	114	45
Responding to survey circulated by Canadian collaboration hub	135	55
Age (years)		
30–39	6	2
40–44	20	8
45–49	61	24
50–54	84	33
55–59	54	21
60+	22	9
Level of physical activity (min/week)		
Active (150+)	128	51
Fairly active (30–154)	85	34
Less active (< 30)	36	14
Receiving treatment for the MT		
Yes (of which complementary and alternative medicines)	108 (7)	43 (3)
No	128	51
Prefer not to answer	13	5
Gender		
Woman	247	99
Agender	1	< 1
Gender fluid	1	< 1
Prefer not to answer	3	1
Geographical spread of responses (country)		
Canada	61	24
New Zealand	41	16
USA	24	10
UK	116	46
Ireland	1	< 1
Singapore	1	
Australia	1	
Greece	1	
UAE	1	
South Africa	1	
Prefer not to answer	1	

*Note:* For the survey distributed from the Canada hub, this list was adapted from Statistics Canada demographics survey options for ethnicity. For the UK, the list reflects the categories used by the Office of National Statistics.

## 3 Using Facet Methodology to Think Through Rhythm

In orienting ourselves to the analysis of the data, we were guided by *facet methodology* (Mason 2011); an orientation to research, which fosters a sustained interest in the varied ways that methods and approaches produce knowledge, and indeed what kind of knowledge. Aside from being qualitatively‐driven, it is not wedded to any singular form of data collection. Rather than attempting to describe and document all dimensions of a problem in its entirety—a task that often proves unwieldy or overly general—facet methodology enables researchers to work through ‘flashes of insight’ generated via exploration of strategically and artfully constructed *facets* of a problem. In this approach, a gemstone serves as a metaphor for the overall research problem or object of concern (in our case, ‘movement through menopause’). Each side–or facet–of the gemstone represents ‘methodological‐substantive planes and surfaces, designed to be capable of casting and refracting light in a variety of ways that help to define the overall object of concern by creating flashes of insight’ (*p* 75). These illuminations are not exhaustive, but they are revealing—generating insight through difference, variation and intersection.

Facet methodology involves multiple lines of enquiry, and different ways of seeing–characteristics we consider particularly apt at a time when the recent ‘menopause turn’ (Jermyn 2023) is gaining cultural and academic prominence, but often in limited or normative ways (Throsby and Roberts 2023). Mason (2011) identifies a series of defining principles characterising facet methodology, which aligned strongly with the assumptions and intentions of our work. For example, facet methodology is underpinned by an anti‐reductionist, connective ontology. Its emphasis lies in tracing connections and entanglements, rather than layering on new categories or lapsing into reductive binaries such as nature/culture, or in this instance, barriers/facilitators to PA. Furthermore, facets are both methodological and substantive, designed to generate *targeted insight* into pressing questions and puzzles, rather than provide an overview of everything.

Furthermore, our interest in movement through menopause through the facet of rhythm, *builds strategically* and artfully on sociological analyses that takes seriously the interrelationship of space and time, rhythmicity and mobilities in the study of ageing, health and physical activity (e.g., Grenier et al. 2019; Phoenix and Bell 2019; Thorpe et al. 2023; Thorpe 2015). It also situates notions of movement and rhythm within shifting cultural and medical meanings of menopause (e.g., as natural, pathological, empowering or health risk), including the management and regulation of menopausal bodies (e.g., via institutionalisation, medicalisation or lifestyle change) (Foxcroft 2009). Developing facets situated within such current knowledge, debates and emerging fields, makes this approach distinctive, giving it the potential to unsettle existing categories, asking different questions in places where dominant frameworks feel insufficient.

We took inspiration from French sociologist and philosopher Lefebvre's theory of rhythmanalysis to develop new ways of thinking about movement through menopause. According to Lefebvre, ‘everywhere where there is an interaction between a place, a time and an expenditure of energy, there is rhythm’ (2004, 15). From this perspective, rhythm is the basis of everyday life, defined as ‘the combination of linear movements and cyclical repetitions, which bring together the mechanistic, rational and structural elements of social life, leisure and work with cosmic or organic cycles’ (Meadows 2010, 84).

Yet not all rhythms are equally visible or valued. As Lyon (2019) notes, many rhythms remain *obscured*—either submerged beneath dominant temporalities or rendered imperceptible by normative structures of time and productivity. Such seeming triviality and taken‐for‐grantedness of ordinary life is what often captures the imagination of those practicing rhythmanalysis, who attend to phenomena that might seem concealed (Chen 2018). These obscured rhythms often surface through feelings of friction, fatigue or disruption, particularly when bodily tempos fall out of sync with social expectations. In the context of menopause, such rhythms may manifest through fluctuating energy levels, disturbed sleep, altered routines or shifting relationships to time and space—elements frequently overlooked in health promotion narratives that privilege consistency, control and productivity. Attending to these quieter, messier rhythms allow us to illuminate dimensions of menopausal experience that are otherwise hidden; those that enter into the lived, rather than necessarily entering the known (Lefebvre 2004).

Our analytical strategy aligned with the principles of facet methodology and data interpretation outlined by Mason (2011, 2018), which emphasise the centrality of researchers' agency and creativity in this process. We began with each author separately reading all survey responses, listening to our data, rather than reacting to impulses to force it into categories. We re‐read the responses in‐person together, immersing, familiarising, noting and discussing analytical ideas that provided (flashes of) insight into something beyond *what* was already known. Intuitively, we aimed to trouble existing binary categories relating to PA during this life stage, reconceptualising and shifting prior assumptions about what makes PA possible or challenging during the MT through an intensive focus on the how and why of (moving) menopausal bodies in time and space. Dialogue remained integral to this iterative process as we wrote and sketched key patterns within the data that were guided by our conceptual sensitivities to notions of time–space patterning, embodiment, affect and the environments of everyday life. Approaching our data in this way involved thinking with, reflecting and imagining as we followed threads across participants responses that could offer novel insights into lived experiences of movement through menopause.

## 4 Moving Through Menopause

The menopause has made it really difficult to exercise and some days it is a struggle to get out of bed. Before HRT, (and sometimes with) my body just aches–the joints hurt and are stiff, putting pressure on my feet is painful. It feels like a deep ache, a weary ache which feeds into the tiredness from having to pull it all together all of the time. Some days, I just want to cry with it all—it feels like I am trapped in a really old woman's body and any spark of vitality has gone. If you go to the Doctors, it is just “a side effect of the menopause”; a real fob off without understanding how it impacts on every aspect of life: getting in and out of the car, walking, everything. It’s easier to sit down, to be as comfortable as possible and just let the exercise go. It is too tiring to fight irrational emotions and an aching body at the same time. Brain fog has meant that in some exercise classes I don't feel as able to concentrate on routines. Some days it is a real force of will power to get out there and exercise. My exercise has become less high intensity and more yoga—the stretching helps. I have only had a couple of hot flashes during the day (mostly mine come at night) but both times have been in an exercise class. I could feel the temperature rising, started to sweat, felt very dizzy and sick. I left the class.

(P99_UK_55–59 years_post‐menopause_MHT_fairly active)^2^

We introduce our findings with a single response which became an increasingly familiar tale of how moving (or attempting to move) through menopause was experienced by our participants. Specifically, symptoms commonly associated with their MT (e.g., hot flashes, joint pain, irregular bleeding, fatigue, brain fog, perceived loss of confidence, rise in anxiety and depression and misunderstanding of health/fitness professionals) hindered PA participation. This supports important findings from an emerging literature focusing on PA barriers, facilitators and subsequent motivators, during the MT (McNulty, Lane, et al. 2024; McNulty, Olenick, et al. 2024; Niven et al. 2025). That noted, extending beyond the existing research, this response also illustrates temporal (day/night, before/after MHT), spatial (bed, fitness classes) and relational (health professionals) dimensions of moving through menopause; a bio‐psycho‐social experience where somatic sensations (sore joints, hot flashes and nausea), emotions, moods and ageing imaginaries surfaced repetitively as moments that constituted everyday life—the patterning of which reveals as different forms of rhythm; namely, cyclical and linear. For Lefebvre, cyclical rhythms originate in nature, evident in the seasons, day and night, biology and the body, while linear rhythms arise from social practice and human activity ‘quantified and fragmented time, imposed by technology, industry and consumption’ (Lyon 2019, 25). Our participants' explanations cast light on this dynamic interplay, particularly in relation to *seasonal*, *hormonal* and *circadian* cycles; cyclical rhythms that were prominent in their responses.

### 4.1 Seasonal Cycles

Seasonal cycles came to the fore in our participants' responses as they referred to the impact of changing temperatures on their everyday experiences of menopause symptoms. For example, hot flashes were felt to ‘virtually disappear in winter’, only to ‘return with a vengeance at the start of summer’ (P55_CAN_45‐49 years_perimenopause_complimentary/alternative medicine_active); a ‘brutal’ time, where cooling off was ‘the worst’ and could ‘take forever’ (P26_CAN_40‐44 years_premature menopause_hormone free dietary supplement_fairly active). Our facet approach enabled the dynamic interplay between seasonal cycles and individual cooling/warming practices to surface. One participant wrote about purposefully drinking more water ‘especially during summer months’ because of the dehydration headaches she experienced from the heavy sweating that accompanied her hot flashes. This creative adjustment, however, could lead to negative consequences for participants' experiences of PA by further exacerbating the need to urinate. As an example:

I am more likely to wee slightly during exercise–especially in the summer when I need to drink more because of the hot flashes–so I sometimes wear period pants or incontinence pads.

(P36_UK_55–59 years_perimenopause_no treatment_active)

Another participant explained how successful cooling sometimes required certain environments—and indeed activities—to be avoided altogether:

Hot flashes are a nuisance and the sweat all over my face can be embarrassing when I’m out for a run or walking briskly. The hot flashes are unavoidable in warm weather especially. I tend to avoid hot yoga now which I use to love. I exercise less outdoors and swim more instead in the summer months, and power walk in cooler weather.

(P65_CAN_45–49 years_perimenopause_complimentary/alternative medicine_active)

The arrival of cooler, winter months could bring some relief to such vasomotor symptoms (i.e., hot flashes). One participant recounted the alleviation that came with being able to ‘go outside in the winter and expose my chest to the cold air’ or, when woken by ‘an intense burning—anything between 20 to 30 times a night—feeling happy to get up and walk around on the cold floor’ (P117_CAN_50–54 years_post‐menopause_MHT_active). It allowed for the removal of layers to ‘control temperature’; a welcomed option that was often unavailable with summer outfits.

In the heat of summer, there is not much more I can do to cool down because I am already wearing light clothing. In the cold of winter, it is possible to remove a layer of clothing.

(P68_CAN_50–54 years_post‐menopause_no treatment_active)

Our analysis highlighted how women's intensified sensations of heating and cooling during the MT prompted creative adjustments relative to clothing; choices regarding what clothes to wear or take along became more meticulous; carrying discarded layers became a norm. Indeed, in pursuit of temperature regulation, the ‘on‐off‐on’ of layering took on its own rhythmicity, which surfaced the opening of intra‐actions between human and non‐human; seasonal weather‐worlds, moving muscles and clothing technologies (wicking and warming).

Expanding what has typically been an almost exclusive focus on heat/hot flashes within menopausal bodies, our analysis provided a ‘partial, angled, illumination’ of participants' embodied experiences of cold (Mason 2011, 80), which often presented as muscle and joint pain. For example:

The trouble with menopause (there are many troubles!) is that you cannot regulate your temperature as you could before menopause. When I get cold, I get really cold, and I guess I tense more, which doesn’t help my muscles and joints

(P61_UK_50–54 years_perimenopause_MHT_active)

Pain is a frequently overlooked and under‐addressed aspect of the menopausal experience (Strand et al. 2025), with raised awareness of ‘musculoskeletal syndrome of menopause’, needed among clinicians and the women they care for (Wright et al. 2024). Through our facet approach, we observed how changeable (non‐human) weather elements intra‐acted with hormonal fluctuations, shifting intensities of pain and stiffness within routinised behaviours and movement practices. Winter months were noted as being ‘much harder’, with sore and aching joints ‘much worse during the colder weather’ (P41_UK_45–49 years_perimenopause_no treatment_active). One participant noted the ‘difficulties exercising outdoors when it is cold, as this exacerbates joint pain’ (P75_UK_55–59 years_post‐menopause_MRT_fairly active). Another wrote how they found the cold had an impact on the elasticity of her connective tissues and muscles making them ‘tighter and more painful’, concluding ‘I need warmth now to exercise and move freely’ (P74_UK_45–49 years_post‐menopause_MHT_active).

These findings add further context to epidemiological research examining the relationship between weather and chronic pain (Dixon et al. 2019) by showing the entanglement of a seemingly linear rhythm of the MT with the cyclical rhythm of seasonal change. Punctuated by the annual repetition of ‘cold snaps’ and ‘damp days’, menopausal bodies are simultaneously weathered bodies, actively engaging in somatic, sensory and weather learning (Allen‐Collinson 2018) to develop embodied knowledge of what and how meteorological conditions become both tonic (cooling) and troublesome (pain inducing), thereby shaping experiences of PA. Shedding light on the cyclical rhythm of seasonal change represents an important early step in integrating menopause research into gendered analyses of climate change (Smith et al. 2020).

Our facet approach offered further insight into the known challenges to mental health during the MT by illuminating its entanglement with seasonal cycles (see Brown et al. 2024). While struggles with mental health were reported to be alleviated by ‘more optimism in the summer’, they were further amplified by ‘feeling general malaise in the dark days’ (P68_UK_50–54 years_perimenopause_MHT_fairly active), with ‘tiredness and anxiety seeming to get worse in the winter’ (P24_UK_55–59 years_post‐menopause_no treatment_fairly active). In their pioneering work on Seasonal Affective Disorder (SAD), Bodden et al. (2024, 598) reinforce the importance of ‘seasonal experience in relation to human well‐being and ways‐of‐being in the world’. Indeed, our experiential weather‐worlds, notes Bell et al. (2019), can make winter negatively impactful for those with limited access to quality light. Such negative impacts were identified in our participants responses, often being articulated through their accounts of tiredness and anxiety. Especially poignant here were heightened feelings of vulnerability and uncertainty around the physical capabilities:

I feel since becoming menopausal I am more worried about being out alone in the dark, I feel on edge if someone approaches and wonder if I would be able to summon enough reserve to run away

(P43_UK_50–54 years_perimenopause_MHT_active)

We refer to this as *worrisome darkness*; where cyclical rhythms of seasonal light provided a context within which menopause symptoms (in this instance, anxiety and fatigue/energy depletion) worked in tandem to shape affective and embodied experiences of movement. This ‘worrisome darkness’ was temporal and spatial, limiting where and when participants felt able or safe to be active. Through our facet approach, we glimpse how the MT is not experienced in isolation but continually modulated by seasonal atmospheres, generating shifting terrains of possibility, constraint and negotiation for movement, rest and wellbeing.

### 4.2 Hormonal Cycles

Shifting temporal framings from the annual to the monthly, our facet approach highlighted hormonal cycles as a further cyclical rhythm patterning participants' experiences of movement through menopause. These were often intertwined with participants' experiences of linear rhythms, which took the form of discrete episodes of brain fog, anxiety or ‘feeling jittery with some clumsiness’ across their cycles. These fragmented episodes might come and go, but there was a persistence, a predictability to them.

Many participants monitored their menstrual cycle with an attentiveness to specific weeks and days, demonstrating insightfulness of how the patterned rhythms of their hormonal cycle could influence physical performance. ‘I went to my usual training session’, reported one participant, ‘I noticed that I was not as sharp as I had been the previous week. I made clumsy errors and could not catch the ball as well as I can when I haven't had those experiences (brain fog, dizziness) in the day’ (P93_UK_45–49 years_perimenopause_MHT_active). Some of those who monitored precisely *when* such time fragments (experienced as episodes of felt symptoms) typically punctuated their typically monthly menstrual cycle, explained how this influenced their PA plans. ‘If [menstrual] periods are too heavy [bleeding] then I can't go to the gym without having a break every 15 mins to visit the toilet. Definitely no swimming at this point in my cycle’ (P88_UK_45–49 years_perimenopause_no treatment_active), wrote one participant. Others offered further detail when describing what considerations were needed prior to being active:

I need to know I'm ok with joints, before a mountain hike or Zumba class as they’ve become very painful with perimenopause. I monitor this all week when I know I'm planning something like a mountain hike. Also, I need to not be on my period (breakthrough bleed) as there is so much blood. I can't Zumba or mountain climb, and I barely go to work when the period happens.

(P64_UK_50–54 years_perimenopause_MHT_fairly active)

Self‐tracking during the menopause transition was not a focus of this research, and subsequent work will no doubt benefit from the rich literature on women's experiences of menstrual tracking apps (e.g., Friedlander et al. 2024; Riley et al. 2025). That said, our analysis highlighted how participants were carefully noticing the temporal patterning of their symptoms (e.g., jittery, clumsy, painful and bleeding) and mapping them onto phases of their hormonal cycle. There is resonance between these practices of *noticing* and *mapping* with the experiences of fragmentation and quantification of time inherent to Lefebvre's linear rhythm. Likewise, with respect to Andelsman’s (2021, 54) observations of how bodies emerge in period‐tracking practices in ways that ‘(re)configure the menstrual cycles as either a series of phases, or an interval with a certain (normative) duration’.

Indeed, these repeated patterned practices of *noticing‐mapping‐planning* (‐for‐PA) (e.g., whether to mountain climb, or Zumba, to swim—or not), contributed to our participants dis/assembling of self‐knowledge and was seemingly integral to their (un) learning of *being*‐(physically active)‐in‐the‐world, during their menopause transition. Such dis/assembling ‘reflects the co‐existent enactment of the self‐as‐known and the self‐as‐not‐known within a specific regime’ (Bergroth 2019, 191). This co‐existence of knowing and not‐knowing, of certainty and uncertainty, was often exacerbated in relation to hormonal cycles through an arising discordance within the (approximate) monthly rhythms of menstrual cycles; a sense of feeling out of step, which Lefebvre refers to as *arrhythmia*.

Many of the written responses illustrated how menstrual arrhythmia disrupted pre‐existing routines that had previously facilitated sustained engagement in PA. As one participant stated: ‘Irregular and heavy periods limits activity, limits the planning of it in the future and prevents some activities’ (P38_UK_45–49 years_perimenopause_no treatment_less active). Similar explanations included:

I have gone from regularly exercising four times per week and walking the dogs daily to some weeks doing nothing. As my monthly cycles are now so irregular and can be heavy, I can feel exhausted. I still pay for my weekly online classes, but some weeks I’ve paid and not attended one.

(P5_UK_40–44 years_perimenopause_seeking MHT_active)

Reference was often made to largely unused gym memberships or missed exercise classes that had required pre‐paid block booking. These comments signalled the rigid, highly structured (timetabled), profit focused fitness industry, pulsing to the implicit (linear) rhythms of weekly, monthly and annual financial targets. They also illuminated the inadequacies of neoliberal agendas for those navigating the new rhythms and routines of their MT; patterns that were increasingly characterised by irregularity and unpredictability.

At the root of participants' apprehension around the irregularity of their menstrual cycle, was an awareness that menstrual bleeding could begin unexpectedly. This had implications for participants' temporal and spatial experiences of PA. For example, while some responded to such uncertainties by ‘only doing exercise in the home’ (P8_UK_45–49 years_perimenopause_MHT_fairly active), others moved in and through more public spaces—albeit ever conscious of an imperative to take necessary precautions to accommodate and at times ‘protect’ against the presence of bodily fluids.

Irregular periods—still waiting for mine to arrive this month. Now they can appear anything from 25 to 40+ days after the last one started. So, I don’t ever want to go out of the house without protection to do my exercise.

(P8_UK_as above)

These comments exemplify consequences of the ongoing subordination of body fluids (be that menstrual fluid, or sweat in the instances of hot flashes), to that which is solid. Our analysis showed how normalised a change to routine, the avoidance of public space and/or the wearing of period ‘protection’ products had become to our participants in response to an increasingly irregular menstrual cycle. This practice of ‘menstrual etiquette’ argues Green‐Cole et al. (2020, 788) creates assumptions that ‘a leaky body is unnatural and only when it is concealed is the body acting normally’. In this way, the perimenopausal, arrhythmic, leaking body is encountered—codified even—as a body that must be managed, contained and often hidden.

### 4.3 Circadian Cycles

Circadian rhythm is understood as a cycle of wakefulness and sleep, ‘naturally’ linked to the cycles of light and dark. Alternations between diurnal and nocturnal rhythms—characterised by human activity and inactivity respectively—conjoin with organised tasks of work, leisure, movement and rest to construct structures and patterns of everyday life (Meadows 2010). Our creative deployment of rhythmanalysis showed how these structures and patterns were frequently destabilised due to participants' arrhythmic accumulations of wakefulness throughout the circadian cycle, experienced as insomnia.

Insomnia is a frequently reported symptom associated with menopause (Proserpio et al. 2020). Our participants' written accounts of ‘sleepless nights’ leading to ‘constant tiredness’ and ‘sheer exhaustion’ featured repetitively in our data. By thinking with rhythm, we were able to move beyond this observation alone, to develop a deeper understanding of participants' articulations of movement, stasis, flow and repetition relative to wakefulness and sleep. Indeed, qualities of sleep described by our participants ranged from initial difficulties getting to sleep, to repeated interruptions to the sleep cycle, which left them ‘tossing and turning in bed’ *all night* (P109_50–54 years_post‐menopause_treatment status unknown_active). For others, discordance was experienced as waking before dawn, ‘*usually around 4a.m.* and can't get back to sleep *for a couple of hours*’ (P42_UK_55–59 years_post‐menopause_MRT_active), sometimes drifting back to sleep just as the alarm clock sounded to signal the transference between Lefebvre's ‘differentiated time’ of sleep and activity. These different arrhythmic scenarios draw into focus the ‘negation of the circadian rhythm’ (Meadows 2010, 88), producing discordant bodies at odds with repetitive patterns of everyday life, including those fundamental to capitalist society, which pivots on the notion of falling asleep in the evening, and awaking at dawn refreshed and ready for activity/work. Conversely, interrupted sleep meant taking ‘a long time to “get going” in the mornings’ (P122_CAN_55–59 yrs_perimenopause_no treatment_active), but it could also result in ‘feeling tired during the day especially in the afternoon’. (P109_UK_50–54 years_post‐menopause_treatment unknown_active).

For Wozniak (2017, 500), arrhythmia can be considered as ‘pharmakon’—both poison and remedy; capable of causing sickness and disorder while also being curative and potentially revolutionary. An arrhythmic circadian cycle—whether experienced as slow mornings, or sleepy afternoons—was met by some participants with new rhythms centred around pacing, re‐scheduling or napping to manage the fatigue that accompanied disrupted sleep. Such creative adjustments, however, could sometimes have unintended consequences as illuminated in this response:

I prefer to exercise early but I find myself more tired in the morning. I sleep poorly now, so I am getting up later and sometimes have little energy to get my day started. If I can get a 30–40 minute nap sometime in the day, it will help with my energy. But if I nap, I end staying up later and then repeat the cycle.

(P67_CAN_55–59 years_postmenopausal_no treatment_active)

Insomnia and disrupted sleep can hinder women's engagement in PA during the MT (Niven et al. 2025). Our findings reinforced this, while also shining light on the *hows* and *whys*. Specifically, they foregrounded the energy cost of living with sleep–wakeful arrhythmia within the circadian cycle to be foregrounded. Rather than seek out practices such as PA that required an expenditure of energy, many participants managed their energy as if a finite resource, proceeding cautiously with its use to ensure they could ‘make it through work’ or ‘get through the day’. While participants rarely elaborated on caregiving or domestic responsibilities, these comments suggest that disrupted circadian rhythms were experienced not in isolation, but against the backdrop of everyday responsibilities and expectations that continued regardless of participants' fluctuating energy levels. Moreover, we noted how deep fatigue disrupted established rhythms of PA, with those who had previously enjoyed early morning activity, sharing how they ‘can't wake up that early anymore’ *(P30_CAN_50–54 years_perimenopause_no treatment_fairly active)*. Reflecting the words of so many, a participant continued, ‘This year has been the least active of my life. I'm just too tired and unmotivated’ (P30_CAN_as above). Feeling unable to engage in a desired level of PA due to constant exhaustion during waking hours evoked a range of affective responses.

The thought of going for an early morning run used to thrill me, now it just fills me with dread: I'll be tired, sore and drag myself through a few miles–when I could be sleeping instead.

(P41_CAN_55–59 yrs_post‐menopause_no treatment_fairly active)

In addition to dread or nervousness about exercising, numerous participants described heightened feelings of anxiety as they lay awake through the night, fully aware of the ramifications it would have for their ability to function the following day; not just in terms of ‘productivity’ or PA, but also ‘in a happy, healthy way’ (P25_CAN_55–59 years_post‐menopause_ alternative therapy_fairly active). Others expressed a sense of defeat and despondency, further drained by ‘the effort of having to push through it all the time’, when ‘all I want to do is sleep and exercise is just a step too far’ (P99_UK_55–59 years_post‐menopause_MHT_fairly active).

Tracing entanglements between (arrhythmic) day–night circadian rhythms, and experiences of PA, it was notable how many of our participants drifted towards what Meadows (2010) identifies as a two‐fold exclusion. That is, exclusion from the ‘natural’ circadian rhythms of sleep *and* wakefulness, and exclusion from the repetitive structure and institutions of everyday life; felt, as the example below indicates, to be the very essence of being in the world; of being human.

Poor sleep. Impacts my ability to function every day. I can’t think/speak/remember and feel. I just want to sleep forever during the day just to feel human.

(P35_UK_50–54 years_post‐menopause_MHT_fairly active)

These findings chime with Lefebvre’s (2004, 75) observation that to become insomniac, is to ‘enter into another everyday‐ness,’ one where both arrhythmia and its manifestation of disrupted sleep is rendered invisible (Meadows 2010). Such invisibility matters because with it, comes incomprehension. Many of our participants were attuned to others limited understanding of their MT, that the (peri) menopausal body was a negotiation of rhythms obscured to many, and consequently rendered inconceivable. Asked what they wished people knew about the impact of symptoms associated with menopause on PA, but is often not talked about, many participants responded with a plea:

I would like more people to understand that women of menopausal age who have decreased their physical activity or seem to be unwillingly to engage in it are not being ‘lazy’. Rather, they could be going through a number of unexpected and challenging symptoms and issues that are unwillingly holding them back. People should be less quick to judge and more understanding.

(P61_45–49 years_perimenopause_no treatment_fairly active)

I wish people understood that we’re not lazy or ‘giving up’—we are just overwhelmed at the changes in our bodies and minds… and it’s unpredictable and scary sometimes, when your body seems to be failing you

(P41_CAN_55–59 years_post‐menopause_no treatment_fairly active)

Invisibility compounds the embodied and affective toll of disrupted sleep, which left many to feel judged or dismissed, as one participant starkly captured:

[I wish people knew] that the fatigue is not funny, I could fall asleep standing up on a bus! That the mental health impact—anxiety, depression is very real and difficult to overcome, so saying ‘just get out there and do it’ really isn’t helpful or supportive.

(P35_UK_50–54 years_post‐menopause_MHT_fairly active)

Such comments offer valuable lessons for health promoters advocating for regular, sustained PA during the MT. While undoubtedly well meaning, such efforts can lead to inactivity being associated with stigma and shame, with little regard for the role of slowing and stillness; for health and wellbeing (Arlie et al. 2025; Flemons et al. 2025).

## 5 Concluding Comments

In this paper, we pay heed to the vitalities of rhythms; their capacity to sustain, disrupt and transform experiences of physical activity during the MT. We demonstrate the value of Mason's (2011) facet approach for tracing the multiple, overlapping and shifting rhythms that participants articulated without collapsing these into static categories or oversimplified linear models. Our findings affirm the importance of staying with the messiness and ambiguity of bodies as they age and change, offering a counterpoint to the certainty and containment often demanded by the methodologies and methods of health behaviour research. At a time when PA is frequently promoted as a remedy menopausal symptoms (despite currently limited evidence), and observed inaction/inactivity of (some) women is often attributed to individual competence, opportunity and motivation, facet methodology enabled us to explore rhythms in and around the body, including those that remain concealed or obscured.

By responding to Lefebvre's speculative invitation to think rhythmically, we advance conceptual understandings of this ‘major life event’ in relation to lifestyle medicine (e.g., physical activity), tracing the interplay of linear and cyclical temporalities and revealing how participants' embodied experiences were shaped by polyrhythmic entanglements of hormones, seasons, symptoms and space. This knowledge extends scholarship on menopause across the sociology of health and illness, critical medical humanities, critical menstrual studies and the sociology of sport. Furthermore, by thinking *with* and *through* rhythm in the context of hormonal change, the paper makes an empirically and theoretically rich contribution to the development *hormonal theory* (Ford et al. 2024); where the complex biosocial reality of hormones, and the psychosocial, cultural, environmental and political influences upon them become a starting point of investigation.

Far from being a biological transition alone, through our research we have exemplified and explained how menopause is also profoundly social, temporal and atmospheric, marked by arrhythmias, re‐calibrations and re‐tunings of body, mood and motion. Our participants encountered their symptoms as rhythmic and arrhythmic relations—moments of harmony and discord—between their bodies and the world around them. Through highlighting these relations, we generate insight into the quieter tunes accompanying movement through menopause while opening space for future research. For example, bringing the cyclical rhythm of seasonality into dialogue with notions of movement through menopause acknowledges the role of seasonality for women's wellbeing and ways‐of‐being in the world. It initiates an important early step in connecting sociological research on movement‐through‐menopause with environment–human health scholarship on issues such as thermal comfort (Hitchings 2011), pain and weather (Dixon et al. 2019) and seasonal affective disorder (Bodden et al. 2024), ultimately offering further credence to the gendered impacts of climate and environmental change, and calls for future research to attend to this (Cucinella et al. 2023).

Our findings also carry applied implications, especially for health promotion and physical activity providers. Health research often isolates ‘barriers’ and ‘enablers’ to physical activity—an approach that may be useful for mapping, but is ultimately binary, atheoretical and inadequate for capturing the dynamic and situated experiences of movement during menopause (Haynes and Loblay 2024). Our research points to a need for rethinking these approaches through a rhythmic lens: recognising that PA routines during menopause are shaped by variable temporalities, unexpected bodily intrusions and a need for greater flexibility in where, when and how movement occurs. After all, movement is as much about the slowing down, the getting stuck and the consequences this has for embodied, emplaced experiences (Phoenix and Bell 2019).

Participants demonstrated a high level of embodied knowledge, often crafting their own responsive, rhythmic strategies to navigate symptoms and maintain movement practices. This highlights the value of involving experts‐by‐experience—peri/menopausal women themselves—in designing movement initiatives that are attuned to the full spectrum of menopausal change, rather than offering PA as a universalised, ‘solution.’ As Hvas and Gannick (2008) caution, dominant health‐promotion discourses risk overpromising by positioning PA as a panacea for all menopausal symptoms. We argue instead that PA should be promoted for its broader contributions to health and wellbeing across the life course. This reason alone warrants its continued promotion. During the MT, PA may ease some symptoms (e.g., hot flashes or low mood) but may have limited impact on others (e.g., joint pain, vaginal dryness). It can also do different things, at different times in response to the obscured rhythms and (un)anticipated affects inherent to seasonal, hormonal and circadian cycles. Meaningful support must be based on nuanced understanding and research evidence, not oversimplified messaging. Drawing from McCormack (2013), in working with the (obscured) rhythms, the task of researchers—and indeed health practitioners—is an ethical one; to multiply possibility for living by attuning more fully to the expressive qualities of movement through menopause.

An important consideration is that while we situated menopause within broader discussions of work, caregiving and gendered expectations in midlife, these dimensions were not a central focus of the survey design. Consequently, participants spoke primarily about bodily, temporal and affective rhythms associated with symptoms rather than the ways these rhythms (inevitably) intersected with responsibilities for paid work, care, household labour and relationship maintenance. Yet traces of these dynamics were evident in accounts of fatigue, depleted energy and the need to conserve resources to ‘get through the day’ or ‘make it through work’. Future research could extend the rhythmanalytic approach developed here by examining how menopausal rhythms interact with the gendered organisation of everyday life and the unequal distribution of care and domestic responsibilities.

This paper contributes to rhythmanalytic scholarship that refuses to reduce menopause to a single story, symptom or solution. Instead, it positions the MT as a polyphonic experience—one that unfolds through melody and beat, dissonance and harmony, across fluctuating temporal scales. Our task, then, is to listen carefully to the rhythms that matter, to foster more eurhythmic spaces for women to move in ways that are responsive and restorative. Yet in so doing, we must remain critically attentive to whose rhythms remain unheard. Despite efforts to diversify our sample, the experiences described here largely reflect those of white, Western, cisgender and university‐ educated women. Notwithstanding the broader context of menopause being under‐funded and under‐researched whereby any advancement of knowledge is welcomed, we acknowledge this group are well represented in much of the research to date; a scenario that can further exacerbate health inequalities experienced by women living with marginalised intersecting identities.

It is in these lived rhythms—both those illuminated in this paper and those still awaiting further attention–that we find the challenge, the potential and indeed the ethical imperative for reimagining movement through menopause: not as prescription, but as attunement.

## Author Contributions

**Cassandra Phoenix:** conceptualization, methodology, data curation, formal analysis, writing – original draft, writing – review and editing, project administration, funding acquisition, investigation. **Meridith Griffin:** conceptualization, data curation, formal analysis, investigation, methodology, project administration, writing – original draft, writing – review and editing. **Erica V. Bennett:** data curation, funding acquisition, investigation, methodology, project administration, writing – original draft, writing – review and editing.

## Funding

This research was supported by Wellcome (Grant No: WT226798/Z/22/Z) and the University of British Columbia's Kinesiology Equipment and Research Accelerator Fund (KERAF). For open access, the corresponding author has applied a CC BY public copyright licence to any Author Accepted Manuscript version arising from this submission.

## Ethics Statement

The research on which this manuscript is based has received ethical approval from the Department of Sport and Exercise Sciences Research Ethics Committee, Durham University (SPORT‐2021‐12‐13T18_33_32‐gjdc83), McMaster University Research Ethics Board (MREB#: 5925), UBC Behavioural Research Ethics Board (PAA #: H22‐00242‐A001).

## Conflicts of Interest

The authors declare no conflicts of interest.

## Permission to Reproduce Materials From Other Sources

The authors have nothing to report.

## Supporting information


**TABLE S1:** Full breakdown of research participants.

## Data Availability

The participants of this study did not give written consent for their data to be shared publicly, so due to the sensitive nature of the research, supporting data is not available.

## Endnotes

^1^
Given our sample, we report here on cisgender women's personal experiences of menopause. However, we acknowledge (some) transmasculine and gender diverse people also experience menopause. When reporting statistics on menopause, we are mindful that available data do not routinely identify gender identity, and that there is a paucity of readily available data on trans and gender diverse experiences of menopause.

^2^
In our findings, we indicate through participant labelling the collaboration hub that the data originates from (i.e., UK or Canada [CAN]). However, because our intention was not to undertake a comparative analysis, in all other reporting both data sets are combined.
