# Supplementary material for: Obscured Rhythms: Using Rhythmanalysis to Understand How Symptoms Associated With Menopause Shape Everyday Experiences of Physical Activity
Source: Sociol Health Illn. 2026 Sep 30;48(8):e70269. doi: 10.1111/1467-9566.70269 (PMC13625026; doi:10.1111/1467-9566.70269)
Supplement: Supplementary file 1 [file SHIL-48-0-s001.docx]

| **Participant characteristic** | **No. of participants** | **% participants** |
| --- | --- | --- |
| Responding to survey circulated by UK collaboration hub | 114 | 45 |
| Responding to survey circulated by Canadian collaboration hub | 135 | 55 |
| **Age (years)**  30-39  40-44  45-49  50-54  55-59  60+ | 6  20  61  84  54  22 | 2  8  24  33  21  9 |
| **Level of Physical Activity (mins/wk)**  Active (150+)  Fairly active (30-149)  Less active (< 30) | 128  85  36 | 51  34  14 |
| **Receiving treatment for the MT**  Yes (of which complimentary and alternative medicines)  No  Prefer not to answer | 108 (7)  128  13 | 43 (3)  51  5 |
| **Identify as Disabled**  Yes  No  Prefer not to answer | 17  213  18 | 7  85  7 |
| **Sexuality**  Heterosexual / Straight  Bisexual  Queer  Pansexual  Gay or Lesbian  Prefer not to answer | 223  12  4  3  4  4 | 89  4  2  1  2  2 |
| **Gender**  Woman  Agender  Gender fluid  Prefer not to answer | 247  1  1  3 | 99  <1  <1  1 |
| **Education Level**  Some high school / high school  Further Education College / Technical / Vocational school  University undergraduate degree / equivalent  Postgraduate education  Prefer not to answer | 29  31  117  69  3 | 12  12  47  28  1 |
| **Ethnic Group***  **(Options in Canada Hub survey):**  European / White  Jewish  South Asian (including sub-continent, Indo-Caribbean, Indo-Fijian, West Indian)  Latin, South of Central American  Indigenous from within North American  Caribbean/Latina  New Zealand Pakeha  East Asian  African/White  European/Maori  European/Non-white  Arab  Pacific  Southeast Asian  East Asian  Middle Eastern  Anglo-Indian  Prefer not to answer  **(Options in UK Hub survey):**  White  Asian / Asian British  Mixed / Multiple ethnic groups  Black / African / Caribbean / Black British  Other Ethnic Group  Prefer not to answer | 116  8  3  2  2  1  1  1  1  1  1  1  1  1  1  1  1  2  106  1  2  2  1  4 | 46  3  1  <1  42  <1  2 |
| **Geographical spread of responses (country)**  Canada  New Zealand  USA  UK  Ireland  Singapore  Australia  Greece  UAE  South Africa  Prefer not to answer | 61  41  24  116  1  1  1  1  1  1  1 | 24  16  10  46  <1 |

**Table 1:** Full breakdown of research participants

*For the survey distributed from the Canada hub, this list was adapted from Statistics Canada demographics survey options for ethnicity. For the UK, the list reflects the categories used by the Office of National Statistics.
